# Methyl Viologen@β-Zeolite with Absorption/Fluorescence Dual-Mode and Photo/Chemical Synergistic Stimuli-Responsive Chromism

**DOI:** 10.3390/molecules30132872

**Published:** 2025-07-06

**Authors:** Jingxuan Han, Shaoning Li, Huihui Li, Yu Li, Jiaqiao Qin, Fuxiang Wang, Qinhe Pan

**Affiliations:** 1 Key Laboratory of Advanced Materials of Tropical Island Resources of Ministry of Education, School of Chemistry and Chemical Engineering, Hainan University, Haikou 570228, China; 15560867778@163.com (J.H.); 18289247353@163.com (S.L.); liyu12303@163.com (Y.L.); 17880957798@163.com (J.Q.); 2 School of Physics and Optoelectronic Engineering, Hainan University, Haikou 570228, China; fuxiang.wang@hainanu.edu.cn

**Keywords:** stimuli-responsive chromism, absorption/fluorescence dual-mode, photo/chemical synergistic, methyl viologen, β-zeolite

## Abstract

In this work, methyl viologen (MV) was adsorbed into the nanopores of Si/Al H-β-zeolite via cation exchange. The resulting MV@β-zeolite possessed absorption/fluorescence dual-mode and photo/chemical synergistic stimuli-responsive chromism. Owing to the acidic surrounding provided by β-zeolite, the chromism of MV required the synergistic stimuli of UV irradiation and a chemical reductant (such as Na_2_SO_3_). UV irradiation induced single electron transfer from the chemical reductant to MV@β-zeolite, leading to enhanced absorption at 610 nm together with a daylight color change from pale yellow to blue. Meanwhile, the nanopores of β-zeolite inhibited aggregation-caused quenching of MV, enabling MV to emit cyan fluorescence at 500 nm. After the single electron transfer of the chemical reductant under UV irradiation, the cyan fluorescence of MV@β-zeolite was quenched. Additionally, MV@β-zeolite exhibited a short stimulus response time (250 s) and good color change reversibility. These findings in this work provide valuable insights into the design of multi-mode and synergistic stimuli-responsive viologen-based chromic materials, particularly for applications in secure high-throughput information storage, high-level anti-counterfeiting and multi-target multi-mode sensing.

## 1. Introduction

Stimuli-responsive materials refer to substances capable of altering their properties (shape, volume, conductivity and optical characteristics) reversibly or irreversibly upon exposure to external stimuli such as heat, light, pH, mechanical force, electricity and chemical agents [1]. These materials hold broad application prospects in smart devices, chemical sensing, anti-counterfeiting labels and biomedical fields [2]. Stimuli-responsive chromic materials, a subset of stimuli-responsive materials, exhibit prominent changes in optical properties accompanied by visible color variations under external stimuli [3,4,5]. Their advantage lies in providing intuitive and real-time visual feedback without requiring complex detection equipment [6]. With potential applications in sensing technology, anti-counterfeiting, smart windows, ink-free printing and information storage, stimuli-responsive chromic materials have become a prominent research focus [3].

Viologen compounds, referring to N,N′-disubstituted 4,4′-bipyridinium, constitute a class of organic chromic materials. The chromic behavior of viologen compounds originates from their exceptional redox activity [7]. Upon exposure to external stimuli (light, electricity, heat, pressure and chemical reductant), the nitrogen atoms in viologen compounds undergo single-electron reduction. This redox process significantly enhances the molar absorptivity of viologen compounds, resulting in distinctive chromic transition under ambient illumination [8]. Recent advancements in viologen chemistry have propelled the development of diversified chromic material architectures, including viologen derivatives, viologen-incorporated conjugated polymers, viologen-based organic–inorganic hybrid composites, viologen-based polymeric networks and viologen-functionalized ionic liquids [9,10,11,12,13]. These viologen-engineered chromic materials demonstrate exceptional performance metrics such as high optical contrast, rapid switching kinetics and robust cycling stability. These advantageous features position them as state-of-the-art candidates for military camouflage, smart display, information storage, anti-counterfeiting, photonic switch and chemical sensing [14,15,16,17,18,19]. As a consequence, viologen chemistry has emerged as a focal point in the research of functional chromic materials.

Current viologen-based chromic materials are predominantly limited to absorption-mode chromism triggered by single external stimulus (light, electricity, heat, pressure or chemical reductant) [1,20]. For satisfying application requirements such as secure high-throughput information storage, high-level anti-counterfeiting and multi-target multi-mode sensing, it is of paramount importance to develop viologen-based materials with multi-mode and synergistic stimuli-responsive chromic properties.

Molecular sieves are inorganic porous crystalline materials constructed from SiO_4_ and AlO_4_^−^ tetrahedra as primary building units interconnected via oxygen bridges to form three-dimensional ordered frameworks [21]. Molecular sieves possess high specific surface areas and tunable pore sizes, and they are divided into microporous (<2 nm) and mesoporous (2–50 nm) types [22]. The aluminosilicate frameworks of molecular sieves inherently contain negatively charged aluminum sites, which confers excellent cation-exchange capacities to these crystalline materials [23,24]. Molecular sieves possess acidity, which originates from the aluminum atoms in their frameworks. On the one hand, framework aluminum atoms bear negative charges, requiring H^+^ to maintain electrical neutrality and forming Brønsted acid sites. On the other hand, three-coordinated aluminum atoms within the frameworks or extra-framework aluminum species can accept electron pairs, generating Lewis acid sites [25]. Owing to their combination of high specific surface area, well-defined pore architectures, size-selective sieving capability, tunable cation-exchange properties and abundant acid sites, molecular sieves have been exploited as adsorbents, gas storage media and heterogeneous catalysts [26,27,28].

Based on their well-defined pore architectures, studies have demonstrated that molecular sieves hold promising applications in modulating the optical properties of guest materials. When guest materials enter the pores of molecular sieves, the microenvironment surrounding the guest materials is changed, thereby influencing the optical properties of guest materials. This mechanism enables the regulation of optical properties of guest materials [29,30,31]. Based on the capabilities of molecular sieves in optical regulation, a wide range of optical functional host–guest materials have been developed. Chen et al. have utilized the acidic channels of β-zeolite to protonate a D-π-A molecule, which synergistically combines with the J-aggregation effect induced by high loading to achieve fluorescence color modulation from cyan to red [29]. Zong et al. have constructed differentiated microenvironments in Mn^2+^-doped AlPO-5 zeolite to create coordinated/uncoordinated dual-emission centers for confined carbon dots. Coupled with an energy transfer mechanism, this system achieves temperature-regulated tricolor phosphorescence dynamic evolution (blue→green→cyan at 77 K; red→green at 298 K) [32].

**Figure 1:**
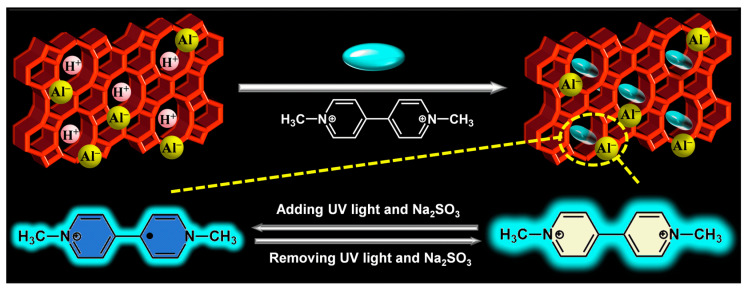
Synthesis and chromic mechanism of MV@β-zeolite.

The purpose of this work was to develop multi-mode and synergistic stimuli-responsive viologen-based chromic materials. Based on diverse properties of molecular sieves, Si/Al H-β-zeolite was here employed to form a host–guest material with methyl viologen (MV) through cation exchange. β-zeolite has a three-dimensional network formed by 12-membered channels with pore diameters of 0.55 × 0.55 nm and 0.76 × 0.64 nm [33]. MV as a linear molecule exhibits a calculated dimension of 1.29 × 0.63 × 0.46 nm [34]. Therefore, the transverse dimension of MV (0.63 × 0.46 nm) is smaller than one pore size of β-zeolite (0.76 × 0.64 nm), which is the reason for the selection of β-zeolite. The stimuli-responsive chromism of MV@β-zeolite was studied. Under the synergistic stimuli of UV irradiation and a chemical reductant (such as Na_2_SO_3_), UV irradiation induced single electron transfer from the chemical reductant to MV@β-zeolite, causing a daylight color change from pale yellow to blue along with quenched cyan fluorescence (Figure 1). The stimulus response time and color change reversibility of MV@β-zeolite were further investigated. This work establishes that the strategic integration of MV with β-zeolite is a rational approach to develop multi-mode and synergistic stimuli-responsive chromic materials.

## 2. Results and Discussion

### 2.1. Characterization of MV@β-Zeolite

**Figure 2:**
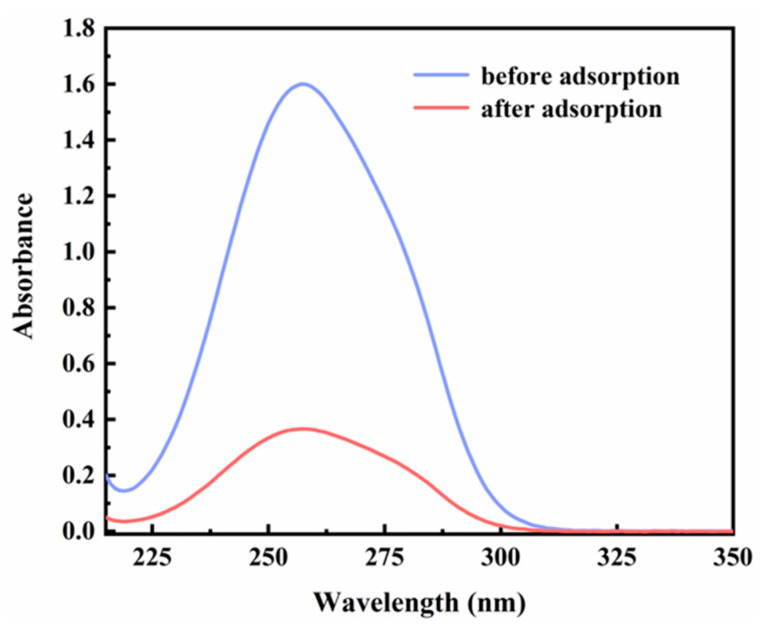
UV-Vis absorption spectra of MV before and after adsorption by β-zeolite.

**Figure 3:**
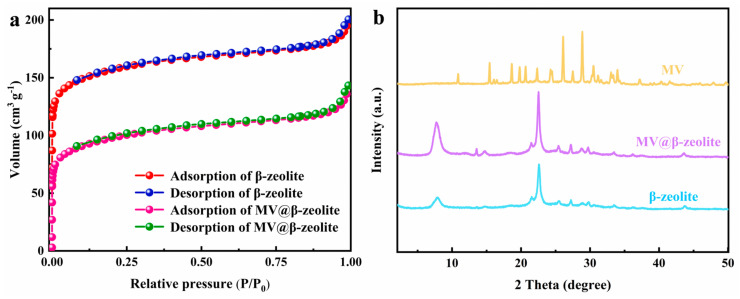
(**a**) N_2_ adsorption tests of β-zeolite and MV@β-zeolite. (**b**) PXRD patterns of β-zeolite, MV@β-zeolite and MV.

**Figure 4:**
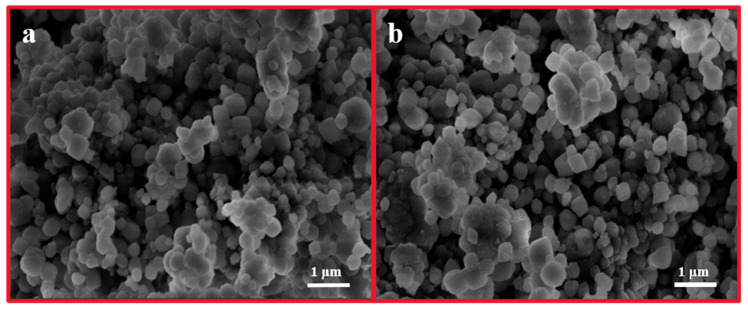
SEM images of β-zeolite (**a**) and MV@β-zeolite (**b**).

In this study, commercially available H-β-zeolite with a Si/Al of 100 was employed as a host material, and MV was incorporated into the nanochannels of β-zeolite via cation exchange to form a host–guest material. To validate the successful preparation of MV@β-zeolite, UV-Vis absorption spectra of MV before and after adsorption by β-zeolite were recorded. As shown in Figure 2, the solution of MV exhibits an absorption peak at 257 nm. However, the absorption peak of the MV solution is reduced after adsorption by β-zeolite (Figure 2), indicating that MV enters the nanopores of β-zeolite. N_2_ adsorption experiments indicate that MV@β-zeolite has a smaller pore volume than β-zeolite (Figure 3a), further confirming that MV enters the nanopores of β-zeolite. Powder X-ray diffraction (PXRD) patterns display identical characteristic diffraction peaks for MV@β-zeolite and β-zeolite (Figure 3b). This result confirms that the crystalline structure of β-zeolite remains intact after the adsorption of MV into its nanochannels. The PXRD pattern of MV was also investigated as a contrast. As validated in Figure 3b, MV@β-zeolite exhibits the same PXRD pattern as β-zeolite rather than MV, which is attributed to the fact that β-zeolite is the host matrix and MV is the guest species. Figure 4a reveals quasi-spherical nanoparticles of β-zeolite by scanning electron microscope (SEM). The SEM image in Figure 4b verifies that MV@β-zeolite maintains quasi-spherical nanomorphology. Therefore, the presence of MV has no effect on the morphology of β-zeolite.

### 2.2. Optical Properties of MV@β-Zeolite

**Figure 5:**
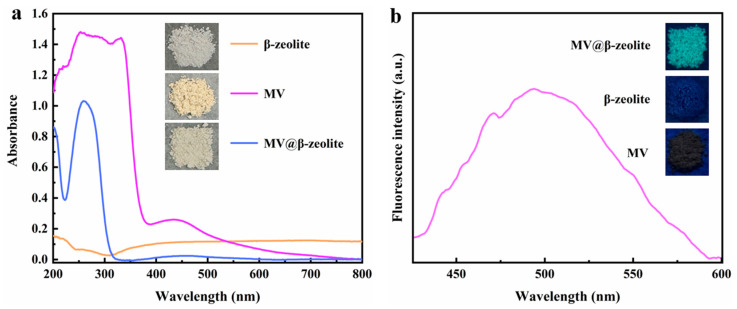
(**a**) UV-Vis diffuse reflectance spectra of solid-state MV, β-zeolite, and MV@β-zeolite; inset: daylight images of solid-state MV, β-zeolite, and MV@β-zeolite. (**b**) Fluorescence spectrum of solid-state MV; inset: fluorescence images of solid-state MV, β-zeolite, and MV@β-zeolite.

The absorption and fluorescence properties of MV, β-zeolite and MV@β-zeolite in solid state were investigated. Figure 5a presents the UV-Vis diffuse reflectance spectra of the three materials. β-zeolite shows no absorption in the UV-Vis region, while MV and MV@β-zeolite both exhibit a strong absorption peak at 257 nm and a weak absorption band at 420 nm (Figure 5a). Correspondingly, β-zeolite, MV and MV@β-zeolite appear white, pale yellow and pale yellow under sunlight, respectively (Figure 5a). Figure 5b characterizes the fluorescence properties of the three materials. Under 365 nm UV light, MV and β-zeolite display no fluorescence emission, whereas MV@β-zeolite emits cyan fluorescence with an emission wavelength of 500 nm (Figure 5b). It can be concluded that MV@β-zeolite possesses a similar absorption characteristic to MV, but the fluorescence properties of MV@β-zeolite and MV are distinctly different.

**Figure 6:**
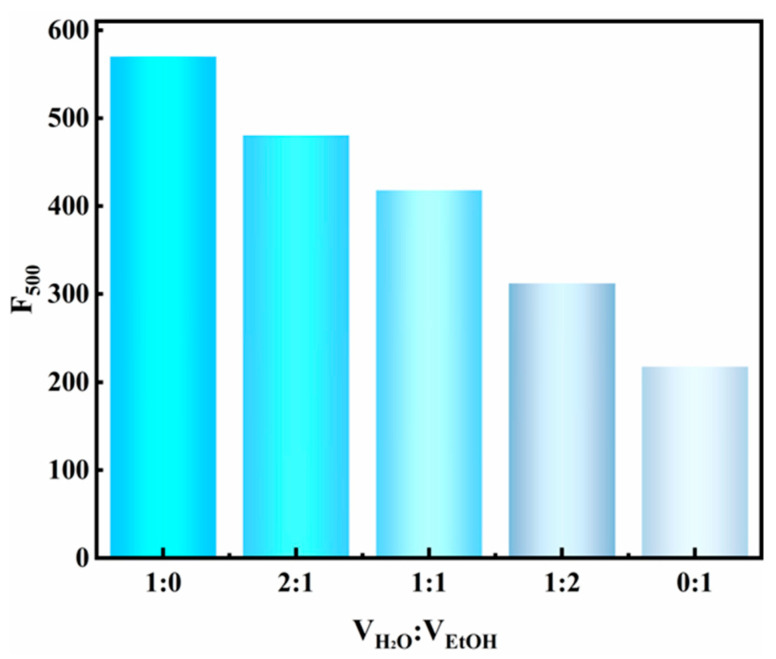
Fluorescence intensities of MV in water–ethanol mixtures with different volume ratios.

Further study was conducted to elucidate the mechanism underlying the differential fluorescence properties between MV@β-zeolite and MV. It is established that most organic molecules exhibit aggregation-caused quenching (ACQ), leading to distinct fluorescence properties between solid powders and host–guest systems. In the solid state, molecular aggregation enhances intermolecular interactions, which facilitates nonradiative decay pathways and quenches fluorescence emission [35,36,37]. When organic molecules are spatially confined within the nanochannels of crystalline porous materials, their aggregation is effectively suppressed, enabling significant fluorescence enhancement [38]. On this basis, we hypothesize that MV exhibits ACQ behavior, which leads to the distinct fluorescence properties between MV@β-zeolite and MV. The verification of the ACQ effect of organic molecules can be achieved by comparing their fluorescence intensities in good and poor solvents. When dissolved in good solvents, organic molecules remain dissolved with strong fluorescence. Conversely, in poor solvents, aggregation of organic molecules induces quenched emission [35]. To demonstrate the ACQ behavior of MV, the fluorescence characteristic of MV in a water–ethanol system was investigated. Water is a good solvent for MV, while ethanol is a poor solvent for MV. As shown in Figure 6, the fluorescence intensity of MV decreases with increasing ethanol content in a water–ethanol system, confirming the ACQ nature of MV [35]. As a consequence, pristine MV in solid state exhibits quenched fluorescence attributed to ACQ, and the nanopores of β-zeolite inhibit ACQ of MV, enabling MV@ to emit cyan fluorescence at 500 nm.

### 2.3. Chromic Property of MV@β-Zeolite

**Figure 7:**
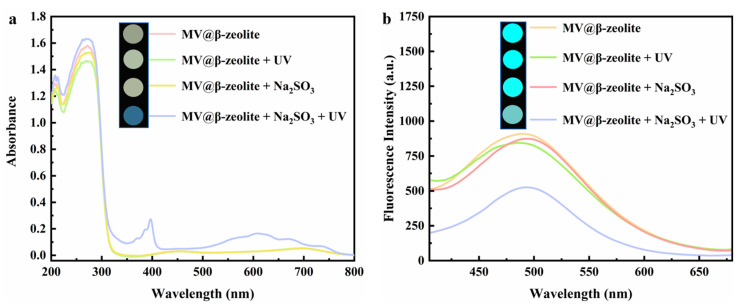
(**a**) UV-Vis diffuse reflectance spectra of MV@β-zeolite before and after the treatment with UV irradiation and Na_2_SO_3_; inset: corresponding photographs taken under natural light. (**b**) Fluorescence emission spectra of MV@β-zeolite before and after the treatment with UV irradiation and Na_2_SO_3_; inset: corresponding photographs taken under UV light.

**Figure 8:**
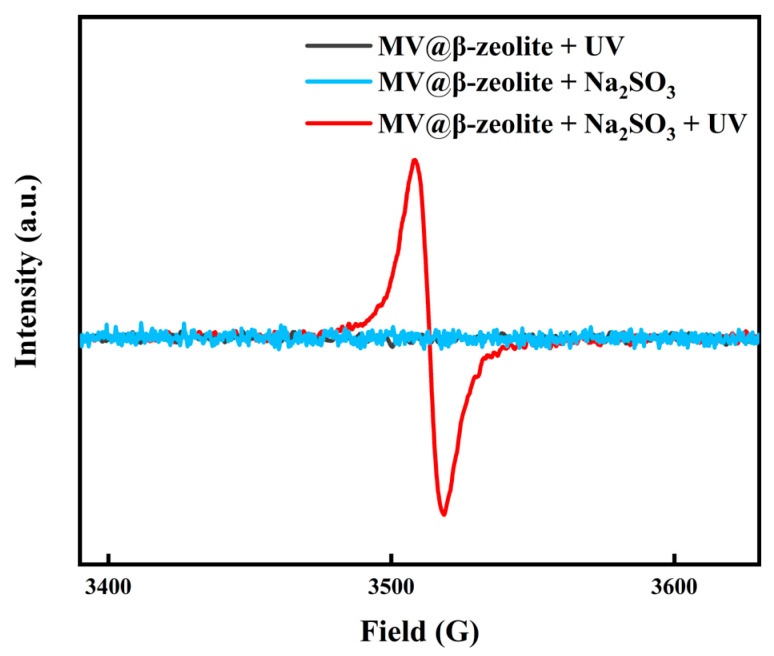
EPR spectra of MV@β-zeolite before and after treatment with UV irradiation and Na_2_SO_3_.

The chromic property of MV@β-zeolite was investigated. Viologen-based materials generally exhibit photochromic properties. Specifically, under light irradiation, single-electron transfer occurs from electron donors to viologen ligands to form viologen radicals, leading to a daylight color change [39,40,41,42]. The electron donors for viologen ligands are typically electron-rich substances, such as polyoxometalate anions, benzenecarboxylate, cucurbit [7]uril, and Co(CN)_6_^3−^ [43,44,45,46]. Figure 7a confirms that MV@β-zeolite exhibits an intense absorption band at 257 nm and a weak absorption peak at 420 nm. Hence, MV@β-zeolite displays a pale yellow appearance under daylight (Figure 7a). As shown in Figure 7b, MV@β-zeolite exhibits a fluorescence emission peak at 500 nm with cyan luminescence. However, after UV light irradiation, no changes are observed in the absorption and fluorescence properties of MV@β-zeolite (Figure 7a,b). Meanwhile, the MV radical is not detected by electron paramagnetic resonance (EPR) (Figure 8). These results indicate that β-zeolite cannot act as an electron donor to undergo a single electron transfer reaction with MV under UV irradiation, failing to change the daylight and fluorescence colors of MV. It has been confirmed that β-zeolite contains Brønsted acid sites and Al-Lewis acid sites [47,48]. Apparently, the acidity of β-zeolite renders it incapable of functioning as an electron donor to undergo a single electron transfer reaction with MV.

**Figure 9:**
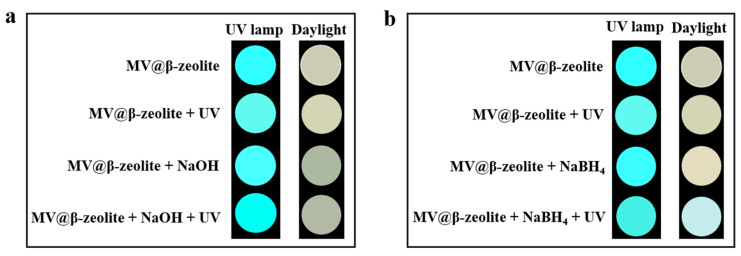
(**a**) Daylight and fluorescence photographs taken for MV@β-zeolite before and after the treatment with UV irradiation and NaOH. (**b**) Daylight and fluorescence photographs taken for MV@β-zeolite before and after the treatment with UV irradiation and NaBH_4_.

To trigger the chromism of MV@β-zeolite, Na_2_SO_3_ as a common chemical reductant was introduced as an electron donor. Figure 7a,b verify that Na_2_SO_3_ cannot alter the absorption and fluorescence properties of MV@β-zeolite, and the MV radical is not detected by the EPR spectrum as displayed in Figure 8. However, under the synergistic stimuli of UV irradiation and Na_2_SO_3_, a new absorption band of MV@β-zeolite at 610 nm is observed, along with a color change from pale yellow to blue under daylight, and the cyan fluorescence emission of MV@β-zeolite at 500 nm is weakened (Figure 7a,b). Accordingly, the MV radical is observed via the EPR spectrum under the synergistic stimuli of UV irradiation and Na_2_SO_3_ (Figure 8). These results confirm that the presence of Na_2_SO_3_ results in the single electron transfer reaction of MV@β-zeolite under UV irradiation, changing the daylight and fluorescence colors of MV@β-zeolite. The role of Na_2_SO_3_ as an electron donor during the chromism of MV@β-zeolite under UV irradiation was further validated. Na_2_SO_3_ not only has a reductive property but also exhibits an alkaline nature. Figure 9a proves that MV@β-zeolite appears pale yellow under daylight and exhibits cyan fluorescence emission. When treated with either NaOH or UV irradiation, MV@β-zeolite maintains unchanged daylight coloration and fluorescence characteristics (Figure 9a). Remarkably, simultaneous stimulation with NaOH and UV light still fails to alter daylight and fluorescence colors (Figure 9a). These results indicate that the reductive property of Na_2_SO_3_, rather than its alkaline nature, drives the chromism of MV@β-zeolite under UV irradiation. To further confirm this conclusion, synergistic stimulation of MV@β-zeolite with an alternative reductant and UV irradiation was explored. Figure 9b shows that MV@β-zeolite retains its original daylight coloration and fluorescence emission when exposed to either NaBH_4_ or UV irradiation. Notably, concurrent stimuli with NaBH_4_ and UV light triggers a chromic transition from pale yellow to blue under daylight and quenched cyan fluorescence (Figure 9b). Therefore, Na_2_SO_3_ serves as an electron donor to undergo single electron transfer reaction with MV under UV irradiation, thus changing the daylight and fluorescence colors of MV@β-zeolite.

### 2.4. Influence Factors of Chromic Behavior

**Figure 10:**
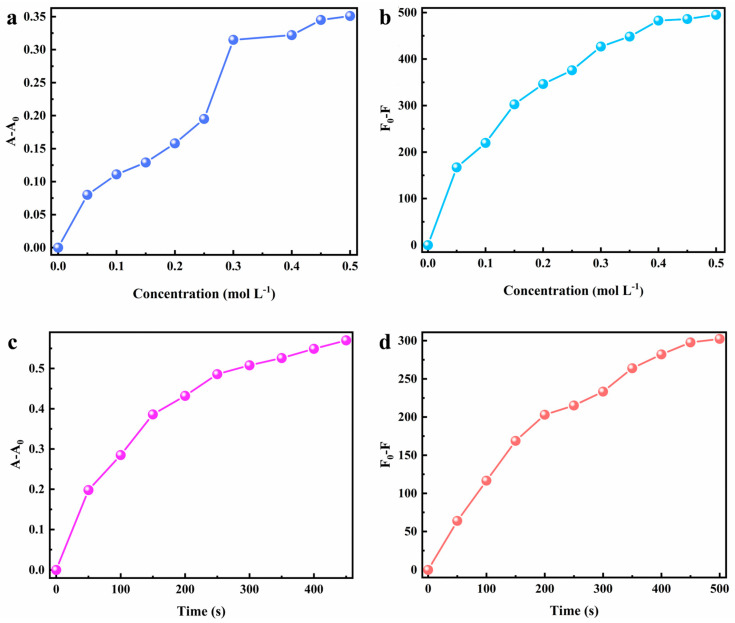
Factors influencing the chromatic property of MV@β-zeolite. (**a**) Effect of the Na_2_SO_3_ concentration on absorption-mode chromism. (**b**) Effect of the Na_2_SO_3_ concentration on fluorescence-mode chromism. (**c**) Effect of UV irradiation time on absorption-mode chromism. (**d**) Effect of UV irradiation time on fluorescence-mode chromism.

The factors affecting the chromic property of MV@β-zeolite were investigated. The stimulating factors for the chromism of MV@β-zeolite include Na_2_SO_3_ and UV irradiation. Therefore, the Na_2_SO_3_ concentration and UV irradiation duration are key factors influencing the chromic behavior of MV@β-zeolite. The impact of the Na_2_SO_3_ concentration on the chromic property of MV@β-zeolite was firstly explored. As the Na_2_SO_3_ concentration increases from 0 to 0.3 mol L^−^^1^, the absorption and fluorescence changes of MV@β-zeolite increase sharply, while when the Na_2_SO_3_ concentration is beyond 0.3 mol L^−^^1^, the absorption and fluorescence changes of MV@β-zeolite increase slowly (Figure 10a,b). Thus, the optimal chromism of MV@β-zeolite is achieved at a Na_2_SO_3_ concentration of 0.3 mol L^−^^1^. Subsequently, the influence of UV irradiation duration on the chromic property of MV@β-zeolite was investigated. As UV irradiation time increases from 0 to 250 s, absorption and fluorescence changes of MV@β-zeolite sharply increase, and after UV irradiation time exceeded 250 s, absorption and fluorescence changes of MV@β-zeolite increase slowly (Figure 10c,d). Therefore, the optimal chromism of MV@β-zeolite is achieved with a UV irradiation time of 250 s.

### 2.5. Chromic Reversibility of MV@β-Zeolite

**Figure 11:**
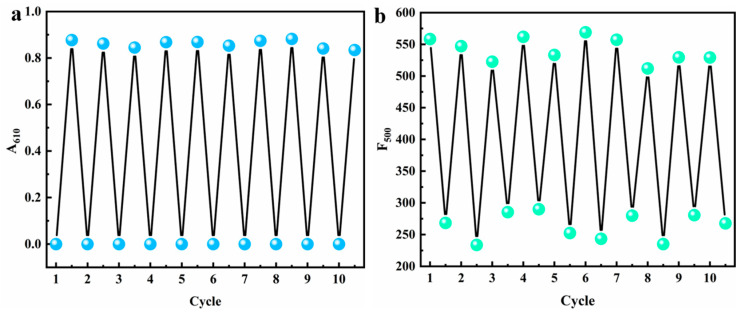
Chromic reversibility of MV@β-zeolite, (**a**) absorption mode, (**b**) fluorescence mode.

In terms of stimulus-responsive chromic materials, reversibility refers to the abilities of materials to return to their initial states upon removal of the external stimuli or through the application of reverse stimuli after undergoing color changes. The reversible chromism of MV@β-zeolite over ten cycles is presented in Figure 11a,b. When the UV light is turned off and Na_2_SO_3_ is removed by centrifugal washing, the daylight and fluorescence colors of MV@β-zeolite are restored to their original states. After stimulation with UV light and Na_2_SO_3_, the absorbance and fluorescence intensity of MV@β-zeolite at the tenth cycle reach 95% and 94% of their initial values, respectively. Thereby, the chromic behavior of MV@β-zeolite possesses excellent reversibility.

A comparative analysis of MV@β-zeolite with other viologen-based chromic materials is shown in Table 1. Reported viologen-based materials respond to single stimulus and only exhibit absorption-mode color change. MV@β-zeolite demonstrates dual stimuli-responsive behavior (UV light and a chemical reductant) with absorption/emission dual-mode chromic phenomenon. Compared with reported viologen-based materials, MV@β-zeolite possesses a more distinctive and versatile chromatic property, showing promising potential for secure high-throughput information storage, high-level anti-counterfeiting and multi-target multi-mode sensing. Most viologen-based materials require over 5 min of stimulation for color changes. By contrast, MV@β-zeolite displays color transition after just 250 s of co-stimulation with Na_2_SO_3_ and UV irradiation. Therefore, MV@β-zeolite displays accelerated response compared to most viologen-based materials. Reported viologen-based materials exhibit excellent reversibility, recovering their original colors upon stimulus removal. Similarly, MV@β-zeolite exhibits reversible behavior, returning to its initial state after the removal of Na_2_SO_3_ and cessation of UV irradiation. Thus, it can be concluded that MV@β-zeolite possesses equally excellent reversibility as other viologen-based materials.

## 3. Materials and Methods

### 3.1. Reagents and Instruments

MV dichloride (CAS No. 1910-42-5) and NaBH_4_ (CAS No. 16940-66-2) were supplied by Macklin Biochemical Co., Ltd. (Shanghai, China). β-Zeolite with H^+^ as the charge-balancing cation at a Si/Al ratio of 100 (CAS No. 12173-28-3) was provided by Zhuoran Environmental Technology Co., Ltd. (Dalian, China). Na_2_SO_3_ (CAS No. 7757-83-7) was obtained from Asahi Kasei Chemical Co., Ltd. (Linyi, China). Ethanol (CAS No. 64-17-5) and NaOH (CAS No. 1310-73-2) were purchased from Xilong Scientific Co., Ltd. (Guangzhou, China). All chemicals were of analytical grade and utilized without purification. Water (CAS No. 7732-18-5) used in all experiments was ultrapure.

Instruments employed included a field-emission scanning electron microscope (Verios G4 UC, Thermo Scientific, Waltham, MA, USA), a powder X-ray diffractometer (MiniFlex600, Rigaku, Akishima, Japan), a specific surface area and pore size analyzer (TriStar II3020, Micromeritics, Norcross, GA, USA), a steady-state and transient-state fluorescence spectrophotometer (FS980, Edinburgh Instruments, Livingston, UK), an ultraviolet-visible spectrophotometer (UV2600, Shimadzu, Kyoto, Japan), an electron paramagnetic resonance spectrometer (A300-10/12, Bruker, Billerica, Germany), an industrial-grade UV flashlight (180 W, 365 nm) to trigger responsive chromism, and a dark-box tri-mode UV analyzer (6 W, 365 nm) for recording fluorescence images.

### 3.2. Synthesis of MV@β-Zeolite

MV (50 mg) was dissolved in water (30 mL) followed by the addition of 500 mg β-zeolite. The resulting mixture was stirred at room temperature for 24 h and then centrifuged (8000 rpm, 4 min) to collect the precipitate. The precipitate was washed five times with water and vacuum-dried at 40 °C overnight to generate MV@β-zeolite. The UV-Vis absorption spectra of the MV aqueous solution were recorded before and after adsorption by β-zeolite. N_2_ adsorption–desorption measurement was performed to analyze the specific surface area and pore structure of MV@β-zeolite. The morphology of MV@β-zeolite was characterized by SEM. PXRD was employed to determine the crystalline structure of MV@β-zeolite. For comparison, pristine β-zeolite was also characterized.

### 3.3. Evaluation of Optical Property

The UV-Vis diffuse reflectance spectrum and fluorescence emission spectrum (Ex = 353 nm) of solid MV@β-zeolite were recorded, with photographic images acquired under daylight and a dark-box tri-mode UV analyzer (6 W, 365 nm). For comparison, the optical properties of pristine MV and β-zeolite were investigated. The UV-Vis diffuse reflectance spectra of solid MV and β-zeolite were recorded, and their fluorescence images were acquired under daylight and a dark-box tri-mode UV analyzer (6 W, 365 nm). To verify the ACQ behavior of MV, water–ethanol mixed solvents with volume ratios varying from 1:0 to 0:1 were prepared. MV was dissolved in these solvent systems (30 mg mL^−1^), followed by the measurement of fluorescence emission spectra (Ex = 353 nm).

### 3.4. Study of Chromic Property

To study photo/chemical synergistic chromism in absorption mode, a Na_2_SO_3_ aqueous solution (20 μL, 0.3 mol L^−1^) was dropped onto the powder of MV@β-zeolite (50 mg), followed by irradiation with an industrial-grade UV flashlight (180 W, 365 nm) for 250 s. The UV-Vis diffuse reflectance spectrum of MV@β-zeolite was recorded, and the photograph of MV@β-zeolite under daylight was captured. To study photo/chemical synergistic chromism in fluorescence mode, a Na_2_SO_3_ aqueous solution (50 μL, 0.3 mol L^−1^) was added to an aqueous suspension of MV@β-zeolite (2.5 mL, 20 mg mL^−1^), followed by irradiation with an industrial-grade UV flashlight (180 W, 365 nm) for 250 s. The fluorescence emission spectrum (Ex = 353 nm) of MV@β-zeolite was measured, and the photograph of MV@β-zeolite in a dark-box tri-mode UV analyzer (6 W, 365 nm) was taken. As a control, the UV-Vis absorption and fluorescence properties of MV@β-zeolite under a single stimulus and without stimuli were also investigated. The operation procedures were the same as described, except for the addition of Na_2_SO_3_, UV irradiation or both.

### 3.5. Research on Chromic Reversibility

To study the reversibility of photo/chemical synergistic chromism of MV@β-zeolite in absorption mode, a Na_2_SO_3_ aqueous solution (20 μL, 0.3 mol L^−1^) was dropped onto the powder of MV@β-zeolite (80 mg), followed by irradiation with an industrial-grade UV flashlight (180 W, 365 nm) for 250 s. The UV-Vis diffuse reflectance spectrum of MV@β-zeolite was recorded. As a control, the UV-Vis absorption property of MV@β-zeolite before stimuli was also studied. The operation procedures were the same as described, except for the addition of Na_2_SO_3_ and UV irradiation. Afterwards, UV irradiation was deactivated, and MV@β-zeolite was subjected to centrifugation washing (8000 rpm, 3 min) with water four times to remove Na_2_SO_3_. After drying at 35 °C in a vacuum oven for 6 h, MV@β-zeolite was regenerated. The above procedures were repeated ten times. To study the reversibility of photo/chemical synergistic chromism of MV@β-zeolite in fluorescence mode, a Na_2_SO_3_ aqueous solution (50 μL, 0.3 mol L^−1^) was added to an aqueous suspension of MV@β-zeolite (2.5 mL, 32 mg mL^−1^), followed by irradiation with an industrial-grade UV flashlight (180 W, 365 nm) for 250 s. The fluorescence emission spectrum (Ex = 353 nm) of MV@β-zeolite was measured. The fluorescence property of MV@β-zeolite before stimuli was also studied. The operation procedures were the same as described, except for the addition of Na_2_SO_3_ and UV irradiation. Afterwards, UV irradiation was deactivated and MV@β-zeolite was subjected to centrifugation washing (8000 rpm, 3 min) with water for four times to remove Na_2_SO_3_. After drying at 35 °C in a vacuum oven for 6 h, MV@β-zeolite was regenerated. The above procedures were repeated ten times.

### 3.6. Exploration of Chromic Mechanism

The formation of the MV radical during the dual-mode chromism of MV@β-zeolite under synergistic stimuli of Na_2_SO_4_ and UV light was investigated. An aqueous solution of Na_2_SO_3_ (20 μL, 0.3 mol L^−1^) was dropped into the solid powder of MV@β-zeolite (50 mg). Subsequently, MV@β-zeolite was irradiated with an industrial-grade UV flashlight (180 W, 365 nm) for 250 s. The EPR spectrum of MV@β-zeolite was then recorded. As a control, the EPR spectra of MV@β-zeolite under a single stimulus of Na_2_SO_3_ or UV irradiation and without stimuli were also measured. The operation procedures were the same as described, except for the addition of Na_2_SO_3_, UV irradiation or both.

The dual-mode chromism of MV@β-zeolite under synergistic stimuli of NaOH (or NaBH_4_) and UV light was studied. An aqueous solution of NaOH (20 μL, 1 mol L^−1^) or NaBH_4_ (20 μL, 1 mol L^−1^) was dropped into the powder of MV@β-zeolite (50 mg) followed by irradiation with an industrial-grade UV flashlight (180 W, 365 nm) for 250 s. The photograph of MV@β-zeolite under daylight was taken. An aqueous solution of NaOH (50 μL, 1 mol L^−1^) or NaBH_4_ (50 μL, 1 mol L^−1^) was added to an aqueous suspension of MV@β-zeolite (2.5 mL, 20 mg mL^−1^), followed by irradiation with an industrial-grade UV flashlight (180 W, 365 nm) for 250 s. The image of MV@β-zeolite in a dark-box tri-mode UV analyzer (6 W, 365 nm) was taken. The photographs of MV@β-zeolite under a single stimulus and without stimuli were also taken. The procedures were the same as described, except for the addition of NaOH (or NaBH_4_), UV irradiation or both.

## 4. Conclusions

Benefitting from the acidity and porous structure of β-zeolite, an absorption/fluorescence dual-mode and photo/chemical synergistic stimuli-responsive chromic material was developed here through the combination of β-zeolite with MV. This work establishes a foundation for the systematic design of multi-mode and synergistic stimuli-responsive viologen-based chromic materials. The resulting MV@β-zeolite exhibits application potential in a wide variety of fields such as secure high-throughput information storage, high-level anti-counterfeiting and multi-target multi-mode sensing. In its current powdered form, MV@β-zeolite demonstrates limited applicability, with its real-world performance remaining uncharacterized. The subsequent research will concentrate on bulk material preparation and practical implementation to advance this work.

## Figures and Tables

**Table 1:** Comparison of MV@β-zeolite with other viologen-based chromic materials.

Material	Stimulus	Mode	Time	Reversibility	References
(MV^II^)_0.5_[Fe^III^Zn^II^(CN)_6_]·(H_2_O)*_n_*	Light	Absorption	30 min	Yes	[49]
{[Cd(cpbpy)(SO_4_)(H_2_O)](H_2_O)_2_}*_n_*	Light	Absorption	15 min	Yes	[50]
CBV-CD	Light or heat	Absorption	7 s	Yes	[51]
[Zn_2_(AQ)_2_(BTEC)(H_2_O)_8_](H_2_BTEC)·6H_2_O	Light	Absorption	5 min	Yes	[40]
Mn_2_(TTVP)(*m*-BDC)_2_	Light	Absorption	60 min	Yes	[52]
MV@β-zeolite	Light and a chemical reductant	Absorption/ fluorescence	250 s	Yes	This work

## Data Availability

The original contributions presented in this study are included in the article. Further inquiries can be directed to the corresponding authors.
